# Communication, continuity and coordination of care are the most important patients' values for family medicine in a fee-for-services health system

**DOI:** 10.1186/s12875-018-0895-2

**Published:** 2019-01-25

**Authors:** Marie Droz, Nicolas Senn, Christine Cohidon

**Affiliations:** Department of ambulatory care and community medicine, Institute of Family Medicine, University of Lausanne, Lausanne, Switzerland

**Keywords:** Family medicine, Values, Communication, Continuity, Coordination

## Abstract

**Background:**

Representing 60% of medical consultations in Switzerland, primary care holds an important place in our medical system. Patients’ values in family medicine (FM) are nowadays recognized as important factors to take into account in order to provide good quality of care. The aim of this study is to describe patients’ most important values regarding FM and to assess their associations with socio-demographics factors in a fee-for-services health system.

**Methods:**

We analyzed the Swiss 2012 study on Quality and Costs of Primary Care (QUALICOPC). Two-hundred patients, randomly drawn, answered a questionnaire about their values regarding FM just after their consultation. Explored values were related to communication and patient-centeredness care, continuity and coordination, care access, and patients’ activation. We described values reaching more than 50% of “very important”. Then, multivariate analyses were performed for the most important value of each dimension.

**Results:**

Items related to “communication and patient-centeredness care” and “coordination and continuity of care” are the most recurrently mentioned as “very important”. Items related to access and patients’ activation are generally declared as “very important” by less than 50% of patients. Whatever the domain and the item, women systematically grant items more often as “very important” than men. Variations are observed according to the age, and the presence or not of a chronic disease.

**Conclusion:**

Such dimensions should be subject to a special attention by general practitioners and public health authorities as it might enhance the quality of care and the patients’ satisfaction.

## Introduction

Representing 60% of medical consultations, primary care holds an important place in our medical system. General practitioners (GPs) assume many different missions, and as a result, meet 90% of patient’s needs [1]. Patients’ satisfaction is nowadays well approved as quality marker of primary care functioning [2, 3], i.e. the more patients are satisfied, the better they will comply with the doctor, leading to greater impact of preventive care, higher continuity of care and, lesser emergency use. In order to promote factual data instead of subjective ones, patients’ satisfaction is usually assessed both through answers to patients’ values and patients’ experiences. Patients’ values reflect the importance that users attach to various aspects of care [3]. These attitudes could be modified by socio-demographics and cultural factors [3–5], as well as healthcare organization. Addressing patients’ values could enhance quality of care, health costs, patients’ behaviours (i.e. adherence to therapy, delay in seeking professional helps) and health outcomes (health status, quality of life) [4, 6, 7]. The literature about patients’ values is somewhat heterogeneous. First, the definition varies among studies. Second, the way to collect data as well as the approach and the content of the questionnaires are divergent [3]. Finally, factors which might influence patients’ values and expectations are inconsistent across studies [3, 8].

The QUALICOPC (Quality and Costs of Primary Care) survey is an international survey which aims to analyze and compare primary care (PC) across different countries [2, 9]. Patients answered questionnaires about their values and experiences related to four dimensions using standardized questionnaires, i.e. (i)care access, (ii) patient activation, (iii) continuity and coordination of care, (iiii) communication and patient centeredness care. As patients’ values may be influenced by the established health system organization, it is of interest to describe these values in a context of fee-for-services system with the free choice of the GP like Switzerland. A perspective with results from other participative countries when available, such as in Canada, may also be interesting [7].

The aim of the present study is to describe patients’ most important values regarding family medicine (FM) in Switzerland and to assess their associations with various patients’ personal and socio-demographics factors.

## Methods

Thirty-one countries took part in the project QUALICOPC conducted by the Nivel Institute from Netherlands to evaluate the primary care system against criteria of quality, equity and costs [2, 9]. The survey included data collection both from GPs and from their patients. In Switzerland, a random sample (from national lists of GPs associations) of two hundred GPs accepted to join the study. For each GP, on a given day at their practice, one patient was randomly selected to answer an auto-questionnaire about his values regarding FM. Data collection was supervised by an investigator just in order to check that the patients answered questionnaires properly.

Survey about patients’ values contained fifty-nine questions; twelve were related to patients’ characteristic and forty-seven to their expectations regarding primary care. To enable comparisons with the Canadian results, items were gathered in the same four dimensions they defined: communication and patient-centeredness care (24 items), continuity and coordination (6 items), access (7 questions), patient activation (10 items) [7]. In a second step, we subdivided the two first dimensions in two sub-dimensions i.e. communication was separated from patient centeredness care and coordination from continuity to improve the accuracy of the result. For each items, patients had to answer if they found the value as “not important”, “somewhat important”, “important” or “very important”. Classical socio-demographic characteristics were collected including area of language (Switzerland is divided into three linguistic areas: German, French, Italian), age, family situation, level of education, language proficiency or skill (Switzerland has numerous foreign communities), income, employment status, origin, mother’s origin. Moreover, global health was measured through two items: perceived health and presence of longstanding illness.

Ethical approval was obtained in accordance with the legal requirements in each country. The Swiss data collection took place between January and June 2012 and was conducted by the Department of ambulatory care and community medicine of University of Lausanne.

For the present study, we focused on the answer “very important” versus the three other ratings as we aimed to evaluate the most important values and to be more discriminative [7]. First patients’ values were rated according to their importance. We also described values reaching more than 50% of “very important”, according to the gender, the existence of chronic disease and the Swiss linguistic area for each dimensions and sub-dimensions. Finally, multivariate analyses were performed to study associations between personal factors and patients’ values. We focused on the most important value of each sub-dimension. Thus, six logistic regressions were conducted separately for each value as dependent variables and patients’ characteristics as independent variables. Variables associated in bivariate analyses at a *p* value of 20% were included in multivariate models in order to build the final model. The analysis was performed using Excel and Stata software.

## Results

Two hundred patients answered the questionnaire. Their median age was 55 years old, 53% were women, 59% were from the German part of Switzerland, 10% from the Italian part and 31% from the French one (Table 1).

**Table 1:** Characteristics of the study sample

Characteristics	N (%)
Gender	
Female	105 (53%)
Male	93 (47%)
Median Age	55
Language	
German	116 (59%)
Italian	20 (10%)
French	62 (31%)
Perceived health	
Poor	18 (9%)
Fair	59 (30%)
Good	89 (45%)
Very Good	32 (16%)
Longstanding disease	
With longstanding disease	76 (38%)
Without longstanding disease	122 (62%)
Other adult in the household	
Yes	131 (66%)
No	66 (34%)
Children in the household	
Yes	40 (20%)
No	158 (80%)
Highest level of education achieved	
No qualifications obtained	55 (28%)
Upper secondary level of education	105 (54%)
Post-secondary, non-tertiary Education or higher	36 (18%)
Language skill	
Not at all	0 (0%)
Poorly	0 (0%)
Moderately	6 (3%)
Sufficiently	16 (8%)
Fluently	175 (89%)
Income (compared to the average income in the country)	
Below average	22 (11%)
Around average	143 (73%)
Above average	30 (15%)
Employment status	
Employed	79 (37%)
Self employed or family business	27 (12%)
Student	10 (5%)
Looking for a job	4 (2%)
Unable to work due to illness or disability	9 (4%)
Retired	70 (32%)
Mainly homemaker	17 (8%)
Country of birth	
In this country	161 (82%)
In an other country	31 (16%)
In Europe	4 (2%)
Mother’s birth country	
In this country	141 (71%)
In another country	51 (26%)
In Europe	5 (2,5%)

Table 2 shows items that reach more than 50% of “very important”. Items related to communication (& patient-centeredness care) and coordination-continuity of care (respectively 10/16 items and 4/16 items rating) are the most recurrently mentioned. Moreover, *“I understand clearly what GP says”*, is the only value that peaks with more than 70% of “very important”. Two items especially related to the sub-dimension coordination of care score more than 50% of “very important”, i.e. *“I know which doctor I will see”* (52%) and “*GP knows when to refer me to a medical specialist”* (68%). Moreover the latter also ranks second across all dimensions. Patient activation is the third dimension represented. However, the two items *“That I adhere to the agreed treatment plan”* (51%) and *“I keep to my appointment”* (50%) reach the lowest score of the top sixteen. None of the seven items belonging to the dimension of access is estimated by more than 50% of patients as “very important”. Patients’ values differ across genders. Although items considered as “very important” are mainly the same for both genders, women generally value 1.5 more highly than men. Results about the existence of chronic diseases are mixed with some unsystematic differences. Higher variations are observed for the items “*GP treats me as a person not as a medical problem*” (70% of “very important” with chronic disease and 58% without) and “*GP takes me seriously*” (71% of “very important” with chronic disease and 58% without). Continuity and coordination of care are systematically valued as more important by patients with a chronic disease (Table 2).

**Table 2:** Items peaking at more than 50% of “very important”, according to gender and existing chronic disease

Item	Sample	Gender			Chronic disease		
	% of total	Male	Female	p	Yes	No	p
I understand clearly what this GP explains	70	59	78	0.04	67	70	0.27
GP knows when to refer me to a medical specialist	68	59	71	0.04	67	65	0.58
GP takes me seriously	64	49	75	< 0.01	71	58	0.06
GP treats me as a person not as a medical problem	64	54	70	< 0.01	70	58	0.10
GP knows information about my medical background	64	57	70	0.07	70	60	0.16
GP listens attentively	63	48	76	< 0.01	68	60	0.22
GP asks questions about my health problem	60	46	72	< 0.01	55	63	0.32
GP is polite	60	43	74	< 0.01	59	60	0.93
GP involves me in making decisions about treatment	57	42	70	< 0.01	51	60	0.25
GP gives me instructions on what to do when things go wrong	54	52	53	0.59	51	53	0.84
GP has my medical records at hand	54	42	65	< 0.01	57	52	0.57
GP asks me if I have any questions	53	37	48	0.08	47	39	0.27
GP does not give me the feeling to be under time pressure	52	39	62	< 0.01	55	48	0.32
I know which doctor I will see	52	42	59	0.02	55	48	0.32
I adhere to the agreed treatment plan	51	38	61	< 0.01	50	50	0.90
I keep to my appointment	50	43	53	0.15	49	48	0.77

The multivariate analyses confirm the importance of socio-demographic features, i.e. gender and age, regarding patients’ values (Table 3). Being a woman (except for items related to access) is systematically positively associated with a higher proportion of “very important”. Patients in middle age place more emphasis on the dimension of coordination (OR = 3.13 [1.41–6.92]) than younger and older ones. The association with middle age class is also high (but not significant) for patient-centeredness care item (OR = 1.93 [0.86–4.35]). Continuity of care and patient-centeredness care are considered as more important among patients with existing chronic disease than others, respectively OR = 1.92 [0.99–3.72] and OR = 2.21[1.05–4.65]. Despite non significant results a good language proficiency is highly associated with the dimension of patient centeredness (OR = 2.84[0.95–8.46]) and continuity (2.40[0.92–6.25]). Finally, none of the considered socio-demographic and personal factors, is predictive of considering access as “very important”.

**Table 3:** Associations between patients’ characteristics and values

Socio-demographic	OR [95% CI] Univariate analysis	OR [95% CI] Multivariate analysis	OR [95% CI] Univariate analysis	OR [95% CI] Multivariate analysis
	*“I understand clearly what GP explains”* (Communication)		“*GP treats me as a person and not as a medical problem*” (Patient-centeredness care)	
Gender				
Male « ref. »	1	1	1	1
Female	2,39 [1.28–4.47]	2.6*** [1.32–5.11]	2.27 [1.25–4.12]	2.75 *** [1.42–5.32]
Age				
< 46 « ref. »	1		1	1
47–64	0.92 [0.41–2.0]		2.32[1.10–4.87]	1.93 [0.86–4.35]
> 65	0.5 [0.23–1.08]		1.08[0.53–2.21]	0.73 [0.31–1.70]
Perceived Health				
Poor and Fair « ref. »	1		1	
Good and very good	1.25 [0.66–2.34]		1.41[0.77–2.60]	
Longstanding disease				
Without « ref. »	1		1	1
With disease	0.83 [0.44–1.54]		1.66 [0.90–3.08]	2.21** [1.05–4.65]
Country of birth				
In this country « ref. »	1		1	
Abroad	1.08 [0.44–2.63]		0.96[0.41–2.23]	
Mother’s country of birth				
In this country « ref. »	1		1	
Abroad	1.5 [0.75–3.02]		0.88 [0.46–1.66]	
Family				
Alone « ref. »	1		1	
With an adult, without children	1.69 [0.85–3.38]		1.31 [0.67–2.58]	
With a child	1.41 [0.60–3.31]		0.93[0.41–2.11]	
Level of education				
No qualifications « ref. »	1		1	
Post secondary	1.49 [0.74–3.02]		1.71[0.67–4.36]	
Upper secondary			0.86[0.44–1.70]	
Language				
Poor level « ref. »	1		1	1
Fluently	1.09 [0.42–2.84]		2.07 [0.83–5.16]	2.84* [0.95–8.46]
Income				
below average « ref. »	1		1	
around	1.65 [0.65–4.15]		.074 [0.28–1.94]	
above	2.27 [0.69–7.55]		0.93 [0.29–3.02]	
Employment status				
Worker « ref. »	1	1	1	
Retired	0.41 [0.21–0.81]	0.36*** [0.17–0.73]	0.78 [0.41–1.5]	
other	1.25 [0.5–3.12]	0.77 [0.29–2.06]	1.43 [0.63–3.25]	
	“*GP knows when to refer me to a medical specialist”* (Coordination of care)		*« GP knows information about my medical background »* (Continuity of care)	
Gender				
Male « ref. »	1	1	1	1
Female	1.72 [0.96–3.09]	1.93** [1.01–3.68]	1.72 [0.96–3.09]	1.91** [1.03–3.56]
Age				
< 46 « ref. »	1	1	1	
47–64	1.65 [0.81–3.38]	3.13*** [1.41–6.92]	1.65 [0.81–3.38]	
> 65	1.27 [0.62–2.59]	1.79 [0.83–3.87]	1.27 [0.62–2.59]	
Perceived Health				
Poor and Fair « ref. »	1		1	
Good and very good	1.32 [0.72–2.41]		1.32 [0.72–2.41]	
Longstanding disease				
Without « ref. »	1		1	1
With disease	1.55 [0.84–2.84]		1.55 [0.84–2.84]	1.92* [0.99–3.72]
Country of birth				
In this country « ref. »	1		1	
Abroad	0.96 [0.41–2.23]		0.96 [0.41–2.23]	
Mother’s country of birth				
In this country « ref. »	1		1	
Abroad	0.97 [0.51–1.84]		0.97 [0.51–1.84]	
Family				
Alone « ref. »	1		1	
FamilyWith an adult, without children	0.66[0.33–1.31]		0.66[0.34–1.31]	
FamilyWith a child	0.74 [0.32–1.70]		0.74 [0.32–1.70]	
Level of education				
No qualifications « ref. »	1		1	
Post secondary	0.57 [0.29–1.14]		1.57 [0.59–4.14]	
Upper secondary	1.57 [0.59–4.14]		0.57 [0.29–1.14]	
Language				
Poor level « ref. »	1		1	1
Fluently	1.92 [0.78–4.68]		1.92 [0.78–4.68]	2.40* [0.92–6.25]
Income				
below average « ref. »	1		1	
around	0.89 [0.35–2.25]		0.89 [0.35–2.25]	
above	1.88 [0.31–0.56]		1.88 [0.56–6.31]	
Employment status				
Worker « ref. »	1		1	
Retired	0.91 [0.48–1.73]		0.91 [0.48–1.73]	
other	2.19 [0.93–5.15]		2.19 [0.93–5.15]	
	*« I adhere to the agreed treatment plan* (Patient’s activation)		*“I can get an appointment easily at this practice” (*Patient’s access)	
Gender				
Gender				
Male « ref. »	1	1	1	
Female	2.67 |1.5–4.77]	2.6*** [1.43–4.71]	1.49 [0.83–2.65]	
Age				
< 46 « ref. »	1		1	
47–64	1.49 [0.75–2.97]		0.86 [0.43–1.73]	
> 65	1.26 [0.62–2.56]		0.96 [0.47–1.96]	
Perceived Health				
Poor and Fair « ref. »	1		1	
Good and very good	1.18 [0.66–2.11]		1.53 [0.85–2.74]	
Longstanding disease				
Without « ref. »	1		1	
With disease	1.04 [0.58–1.85]		1.11 [0.62–2]	
Country of birth				
In this country « ref. »	1		1	
Abroad	0.76 [0.33–1.73]		1.36 [0.59–3.09]	
Mother’s country of birth				
In this country « ref. »	1		1	
Abroad	0.87 [0.47–1.61]		1.27 [0.68–2.37]	
Family				
Alone « ref. »	1		1	
With an adult, without children	1.53 [0.8–2.93]		1.45 [0.74–2.81]	
With a child	0.66 [0.29–1.49]		1.19 [0.52–2.74]	
Level of education				
No qualifications « ref. »	1		1	
Post secondary	1.71 [0.72–4.04]		0.63 [0.27–1.49]	
Upper secondary	0.84 [0.44–1.62]		0.67 [0.34–1.3]	
Language				
Poor level « ref. »	1		1	
Fluently	3.44 [1.2–9.89]		0.63 [0.26–1.54]	
Income				
below average « ref. »	1		1	
around	1.23 [0.50–3.04]		1.22 [0.48–3.10]	
above	1.2 [0.4–3.62]		0.875 [0.28–2.77]	
Employment status				
Worker « ref. »	1		1	
Retired	0.96 [0.51–1.82]		1.09 [0.57–2.09]	
other	1.38 [0.65–2.39]		1.2 [0.56–2.55]	

Multivariate model adjusted on linguistic area

*0.05<*p*<0.01 **0.01=<*p*<0.001 ***p<=0.001

## Discussion

Ten of the sixteen values reaching more than 50% of “very important” belong to the dimension “communication and patient-centeredness care”, and four of them to the dimension “continuity and coordination”. No item regarding access reaches more than 50% of “very important”. Being a woman is systematically associated with a higher importance rate (except for access items) while linguistic skills, age and having a chronic disease are sometimes also associated with patients’ values.

### Comparison with the literature

Communication appears as the dominant dimension in our study, especially regarding the patient-physician relationship during the consultation. Respectful relationship through attentive listening, high consideration and clarity of the information exchanged are the most valued elements by the patients. The importance of communication has already been reported in many previous studies and, in particular, in other QUALICOPC participative countries like Canada and Greece [3, 7, 10]. In 1998, the review of Wensing, including fifty-seven studies about values in primary health care, already reported that communication reached the highest score in 86% of the studies [3]. The critical importance of this dimension in FM seems to persist overtime. Our results regarding access are also consistent with the literature review by Wensing: availability, accessibility and organization were seen by patients as less important [3]. Waiting period was also a minor expectation according to Sebo in 2015, who showed about care access that: “*seeing a doctor of choice*” was more important than “*having an appointment sooner rather than later*” [4]. In Greece, results are mixed since a short distance to the practice is highly valuated whereas the items pertaining to easy appointments are less rated. In Canada, where public health authorities consider PC access as a priority, results were very similar. It is however possible that the study missed some aspects of care access since it included patients who already had access to care. Moreover, even if no item reaches more than 50% of “very important”, they are considered at least as “important” by a majority of patients.

More innovative are the results pertaining to the other dimensions. Two items of continuity were rated with more than 50% of “very important”. Despite the fact that they are different sub-dimensions, coordination is close to continuity. The importance of continuity was also reported from the Greek data ^10^The Canadian study also reported that coordination and continuity were together evaluated as “very important” by more than 80% of their population [7]. In 1998, according to the review by Wensing, half of the studies did not evaluate coordination as an important value [3]. This discrepancy could be due to the aging population. Indeed, doctors and public health planers have nowadays to face up to patients experimenting more chronic diseases [11]. Moreover, the health system is more and more specialized and each patient will have to see many different health professionals [12]. Because of these two major issues, coordination and continuity need to be central points for public health strategies in industrialized countries [13]. They both have also been demonstrated to decrease the emergency use and to enhance prevention and health promotion [7, 13].

Only two of the ten items related to patient activation reach more than 50% of “very important”. Patient activation has been claimed to be associated with an improvement of patients’ behavior, compliance and life quality, ultimately leading to a better health quality, health outcome and patients’ experiences [7, 14]. However, several researches currently show that if, in general, patients want to be informed about choices about their health, a complete shared decision-making is still debated [15]. Greek and Canadian data go in the same direction.

### Predictive factors of patients values

Dimensions evaluated as the most important are the same across gender, but women systematically rate items higher, especially the ones related to communication. One possible explanation is that women systematically receive more information, ask more questions and have more partnership with physicians [16].Having a chronic disease is not surprisingly associated with the dimensions of continuity and patient-centeredness care. As the presence of longstanding disease goes along with patients seeing many doctors, information flows between different medical professionals are even more crucial [17]. Moreover, due to daily confrontation with their sickness, chronic patients need a customized education with regard to their personal way of living [18, 19].

### Limitations

This study has several limitations. First, only 200 patients answered the questionnaire. This could limit the extrapolation of the results to the whole population of patients in FM. However, the participation rate (around 84%) was very good, which limits the risk of selection bias. The data were collected in 2012 that is somewhat old. However, we can reasonably assume that changes in patients’ values are not quickly labile. Finally, the design of the study does not enable to conclude in a causal nature of the observed associations.

## Conclusion

Unlike access and patient activation, the most important value of patients regarding family medicine are communication, continuity and coordination of care. The latter appears nowadays particularly critical because of the increasing complexity of health care delivery. This is even more important for patients suffering from chronic diseases, suggesting that regular contact with GPs increase the importance of a follow-up throughout consultations. Beyond the fact that communication remains a key core of family medicine, it should be no longer just a question of style, that is, politeness and respect of the person; there is now probably also a need for interactivity between patients and providers. In addition, further investigations, especially qualitative studies, could be led in order to better understand the potential positive impact of communication/coordination on the quality of care, beyond patients’ satisfaction.

## Abbreviations

FM: Family medicine
GP: General practitioner
OR: Odds ratio
QUALICOPC: Quality and Costs of Primary Care
